# Risk and contributing factors of ecosystem shifts over naturally vegetated land under climate change in China

**DOI:** 10.1038/srep20905

**Published:** 2016-02-12

**Authors:** Yuanyuan Yin, Qiuhong Tang, Lixin Wang, Xingcai Liu

**Affiliations:** 1 Key Laboratory of Water Cycle and Related Land Surface Processes, Institute of Geographic Sciences and Natural Resources Research, Chinese Academy of Sciences, Beijing 100101, China; 2 Department of Earth Sciences, Indiana University-Purdue University Indianapolis (IUPUI), Indianapolis IN 46202, USA

## Abstract

Identifying the areas at risk of ecosystem transformation and the main contributing factors to the risk is essential to assist ecological adaptation to climate change. We assessed the risk of ecosystem shifts in China using the projections of four global gridded vegetation models (GGVMs) and an aggregate metric. The results show that half of naturally vegetated land surface could be under moderate or severe risk at the end of the 21^st^ century under the middle and high emission scenarios. The areas with high risk are the Tibetan Plateau region and an area extended northeastward from the Tibetan Plateau to northeast China. With the three major factors considered, the change in carbon stocks is the main contributing factor to the high risk of ecosystem shifts. The change in carbon fluxes is another important contributing factor under the high emission scenario. The change in water fluxes is a less dominant factor except for the Tibetan Plateau region under the high emission scenario. Although there is considerable uncertainty in the risk assessment, the geographic patterns of the risk are generally consistent across different scenarios. The results could help develop regional strategies for ecosystem conservation to cope with climate change.

Climate change has a strong influence on terrestrial ecosystems, and the influence is almost certain to grow in the near future^1^. Global warming and the associated rise in extreme temperatures substantially increase the chance of concurrent droughts and heat waves^2^,^3^. Continuing changes and climate extremes could cause significant impacts on the terrestrial ecosystems, such as changes in regional carbon stocks and vegetation leaf cover^2^,^4^.

China has a variety of ecosystems ranging from alpine tundra to evergreen tropics and from desert to forest. The impact of climate change on terrestrial ecosystems in China has attracted considerable interests. Many impact assessments focus on one or a few aspects such as vegetation structure and distribution^5^,^6^, or carbon cycle (e.g. carbon flux and stock, net primary productivity (NPP))^5^,^7^,^8^. Because change in NPP integrates climatic, ecological, geochemical and human influences on the biosphere^9^, NPP has been widely used as an index to quantify ecosystem change in climate change impact assessments^8^. Although NPP is a useful index, it does not explicitly represent the changes between future and present ecosystems in stocks and fluxes of carbon and water, as well as vegetation structures. Thus it is difficult to identify the main factors influencing ecosystem shifts and to know to what degree climate change can be tolerated before complex ecological systems start to shift.

In order to quantify the comprehensive risk of ecosystem changes under climate change, Heyder *et al.*^10^ proposed an aggregated metric, Γ, of joint changes in macroscopic ecosystem features, which have been used in the risk analysis of climate change impacts on the terrestrial biosphere at a global scale^11^,^12^,^13^,^14^. This metric is based on a specific subset of macroscopic variables (e.g., carbon fluxes, carbon stocks and water fluxes) that characterize the ecosystem state. Essentially, this metric Γ uses aggregated changes in the biogeochemical ecosystem state as a proxy for the risk of ecosystem shifts. Ecosystem shift in this study refers to the state that the ecosystem has been pushed beyond the point of recovering^15^. Based on the specific subsets of variables, this metric could also be used to identify the main factors influencing ecosystem shifts. The ecosystem risk under climate change in China has been assessed with the metric Γ in the global analysis, however, the surface covered by natural vegetation was not accurately represented in the global analysis^16^,^17^. Although an administrative area of interest may be extracted from a global assessment, the regional analyses are generally not readily available for decision makers who are more interested in the risk and the contributing factors of the risk at the level of administrative area or eco-regions. The objective of this study is to identify the high risk areas and the main contributing factors of the predicted ecosystem shifts for natural vegetation under climate change in China and to support adaptation to climate change at the regional scale.

In order to identify the high-risk areas and the main contributing factors of the predicted ecosystem shifts at regional scale, we present a multi-model analysis of ecosystem shift risk in China using the metric Γ. The metric Γ was calculated under various climate scenarios^18^ provided by the Inter-Sectoral Impact Model Intercomparison Project (ISI-MIP)^19^. In ISI-MIP, the GGVMs were forced with the bias-corrected climatic variables from four Representative Concentration Pathways (RCPs) outputs of five global climate models (GCMs). It provides an opportunity for assessing ecosystem risks of climate change in the 21^st^ century. We used the simulations of four GGVMs together with the land cover data of China^20^ to calculate the metric Γ in the eco-regions of China and to identify the major contributing factors of the ecosystem shift risk by analyzing the variables contributed most to the change in Γ. Although previous work has provided a global assessment of ecosystem shift risk^11^, there are few attempts to assess the risk at regional scale. Comparing with the global assessments, regional information is more useful to guide adaptive ecosystem management especially for nations with various eco-regions such as China (Fig. 1). The main questions of this study are: i) what are the region (s) with high risk of ecosystem shift under different climate change scenarios in China? ii) What are the main contributing factors of the predicted ecosystem shifts? And iii) what are the major sources of uncertainties in the estimates?

## Results

### Dimensions of ecosystem change

Figure 2 shows the dimensions of changes for carbon fluxes, carbon stocks and water fluxes at the end of the 21^st^ century (2070–2099) under RCP 8.5. The local relative changes (*c*) in carbon fluxes and stocks are large in the northwest arid region and the Tibetan Plateau region, where vegetation carbon and NPP are currently low but would increase in the future due to the effects of temperature rise and CO_2_ fertilization^21^. The local relative changes (*c*) in water fluxes are large in the arid areas where a small amount of absolute change may indicate a large relative change since the overall fluxes are small. The projected absolute changes (*g*) in carbon fluxes, carbon stocks and water fluxes are large in the Tibetan Plateau region, implying this region would experience substantial ecosystem changes in a warming environment. The absolute changes (*g*) in carbon fluxes and stocks are large at the humid regions in eastern China because vegetation carbon, NPP and transpiration are high in the humid regions. The absolute changes (*g*) in water fluxes are small in the arid areas but relatively large in the tropical and sub-tropical humid region in southern China where the projected runoff would decrease^22^,^23^. The changes in the relative magnitude of carbon fluxes, carbon stocks and water fluxes (*b*) are generally small. The result is in line with the spatial patterns in Heyder *et al.*^10^ except for some areas in the Tibetan Plateau region, likely due to the significant uncertainty in ecosystem responses to climate change in different GGVMs. The spatial patterns of the projected dimensions of changes in ecosystem properties are similar for the other RCPs. However, the changes under higher emission scenario are generally higher than those under lower emission scenario (Supplementary Figure S1–S3).

Figure 3 shows the calculated metric Γ for each of the variable subset ‘carbon fluxes’, ‘carbon stocks’, or ‘water fluxes’ individually (Supplementary Table S1) at the end of the 21^st^ century under four RCPs. Depending on the variable subset considered, the spatial patterns of the metric vary greatly. It suggests that the contributing factors to the ecosystem shifts are different for different regions. For a specified variable subset considered, the spatial pattern of the metric under different RCPs is similar although the metric is generally small under the lowest emission scenario RCP 2.6 and becomes greater under higher emission scenario. The metric Γ for carbon fluxes under RCP 8.5 is considerable in the Tibetan Plateau and temperate humid/sub-humid regions where the changes in carbon fluxes are likely caused by temperature-driven increase in soil respiration and CO_2_ fertilization effects on NPP^24^,^25^,^26^. The metric Γ for carbon stocks under RCP 8.5 is greater than 0.3 in the Tibetan Plateau region and the eastern China region. Based on simulations of the GGVMs, the metric Γ for water fluxes is small in most parts of China under all emission scenarios. The only exception is the Tibetan Plateau region where the median of the metric Γ for water fluxes is greater than 0.25 under RCP 8.5. The results are in line with Heyder *et al.*^10^ and Warszawski *et al.*^11^

### Risk of ecosystem shifts

Figure 4 shows the integrated ecosystem change metric Γ combining all the variable subsets (Supplementary Table S1) at the end of the 21^st^ century under four RCPs. Under the lowest emission scenario RCP 2.6, the ecosystem change metric Γ is small in most parts of China except for an area in the Tibetan Plateau region, where the ecosystem shifts are moderate or severe. The area with severe or moderate risk of ecosystem shifts becomes larger under higher emission scenarios. Under RCP 8.5, the Tibetan Plateau region and part of the temperate humid/sub-humid and cold temperate humid regions would have severe risk of ecosystem shifts. The high value of Γ over the Tibetan Plateau region results from the combination of the changes in carbon fluxes, carbon stocks, and water fluxes (Fig. 3). The temperate humid/sub-humid and cold temperate humid regions would have moderate risk of ecosystem shifts. With the three major factors considered, the changes in carbon fluxes and carbon stocks are the main contributing factors to the risk of ecosystem shifts (Fig. 3). The risk of ecosystem shifts is generally low in the northwest arid region and tropical and sub-tropical humid region. In these very dry or very wet regions, the projected changes in water fluxes and carbon fluxes show relative small contribution to ecosystem shifts (Fig. 3). More than 50% of the model pairs suggest severe risk of ecosystem shifts over one part of the Tibetan Plateau region under RCP 2.6 (Supplementary Figure S4). The consistent model agreement on the risk assessment indicates the ecosystems in the Tibetan Plateau would be strongly affected by climate change even if the required emission reduction rates is achieved to keep global mean temperature increase below 2 °C^27^. The model agreement on the severe risk estimates is generally low over other regions of China under RCP 2.6. Over the Tibetan Plateau region, the area with high model agreement on severe risk becomes larger under RCP 4.5 and RCP 6.0. Moreover, the area with high model agreement on severe risk extends northeastward to the temperate humid/sub-humid region and cold temperate humid region (Supplementary Figure S4). Under RCP 8.5, most model pairs suggest severe risk over a large part of the temperate humid areas at the end of the 21^st^ century (Fig. 4). The area with the severest risk is in the southern and eastern Tibetan Plateau. In the global analysis, the area with the severest risk is also over the Tibetan Plateau although the high-risk area is in the north^11^. The risk in warm temperate humid/sub-humid regions is higher than that in the global analysis^11^. Possible reasons could be that the globally averaged changes are replaced with regional specific values in China, and that the metric of change in vegetation structure is not included in this calculation.

Figure 5 shows the area fractions with severe and moderate risk of ecosystem shifts under different RCPs from the present to the end of the 21^st^ century. The area with severe risk is generally small (less than 5%) before 2030 (i.e., the period of 2016–2045) under all RCPs. Under RCP 2.6, the fraction of area with severe risk reaches 5% at 2050 and increases slightly thereafter. The fraction of area with severe risk exceeds 5% at 2035, 2040, and 2050 under RCP 8.5, RCP 4.5, and RCP 6.0, respectively. The fraction of area with severe risk under RCP 6.0 is smaller than that under RCP 4.5 before 2070, likely because the greenhouse gas (GHG) concentration under RCP 4.5 is generally higher than that under RCP 6.0 before the mid-21^st^ century^28^. At about 2070, the area with severe risk under RCP 4.5 and RCP 6.0 occupies 15% of naturally vegetated area while the fraction of area with severe risk under RCP 8.5 is 27%. At the end of the 21^st^ century, the fraction of area with severe risk reaches 17%, 23% and 35% under RCP 4.5, RCP 6.0, and RCP 8.5, respectively. The fraction of area with severe risk under RCP 6.0 becomes larger than that under RCP 4.5 at the end of the 21^st^ century as the GHG concentration under RCP 6.0 becomes larger in the late 21^st^ century^28^. The area with moderate risk is much larger than that with severe risk. At the end of the 21^st^ century, about one quarter of the area would face moderate risk of ecosystem shifts under RCP 2.6. The fraction of area with moderate risk under RCP 6.0 is smaller than that under RCP 4.5 at the beginning but becomes similar after 2070. The area with moderate risk under RCP 6.0 and RCP 4.5 occupies about half of the area by the end of the 21^st^ century.

**Figure 1:**
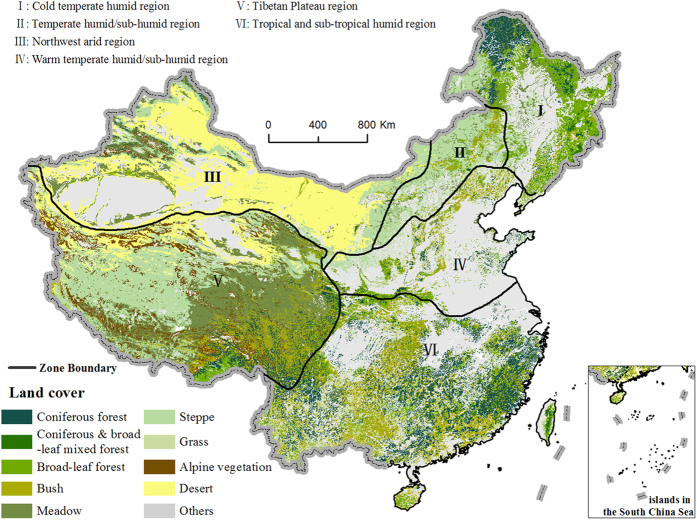
Main land cover types and six eco-regions in China. The cultivated area, sandy desert and swamp are not included in the analysis and are classified as others in the map. The boundary of the regions is adopted from Zheng^27^. The land cover data were obtained from Compiling Committee of Chinese Vegetation (CCCV) with the scale of 1:1 000 000^17^. We generate the figure using ArcGIS software (http://www.arcgis.com).

Figure 6 shows the mean, median and inter-quartile range of the integrated Γ estimates of all three variables in the six eco-regions of China^29^ under different RCPs at the end of the 21^st^ century. Under RCP 2.6, the median of the Γ estimates for all variables in the Tibetan Plateau region is 0.1, indicating that half of the grids in the Tibetan Plateau region would have moderate risk of ecosystem shifts. Less than 25% grids in the Tibetan Plateau region would have severe risk while the risk is below the severe level for all the grids in the other regions. The medians of the Γ estimates in the Tibetan Plateau region are greater than 0.1 for both carbon fluxes and stocks but less than 0.1 for water fluxes, suggesting that with the three major factors considered, the changes in carbon fluxes and stocks are the main factors contributing to the moderate to high risk. Although the Γ estimates for carbon stocks are greater than 0.1 for most grids (more than 75%) in the humid and sub-humid regions (regions I, II, IV, V (Fig. 1)), the Γ estimates for carbon and water fluxes are generally small. As a consequence the integrated Γ estimates are less than 0.1 in these regions. The Γ estimates are small for each variable subset and for the integrated Γ estimates over the northwest arid region, indicating a low risk of ecosystem shifts for this region.

Under RCP 4.5 and RCP 6.0, more than 25% grids in the Tibetan Plateau region would have severe risk and more than 75% grids would have moderate risk of ecosystem shifts. The Γ estimates for both carbon fluxes and carbon stocks are greater than 0.3 at more than half of the grids in the Tibetan Plateau region, indicating that with the three major factors considered, change in carbon is the main contributing factor to the high risk of ecosystem shifts. Under RCP 6.0, more than 50% grids in the humid/semi-humid regions (regions II and IV) would have moderate risk, largely due to changes in carbon stocks and fluxes. The change in carbon stocks is large, with Γ for carbon stocks greater than 0.1, over the cold temperate humid region (region I) and tropical and sub-tropical humid region (region VI). However, the changes in carbon and water fluxes are small with the corresponding Γ value less than 0.1 in these regions. This makes the integrated Γ estimates are below the moderate risk level at more than half of the grids in regions I and VI.

Under RCP 8.5, Γ estimates for carbon stocks and fluxes are greater than 0.3 for more than half grids and Γ estimates for water fluxes are greater than 0.3 at about half grids in the Tibetan Plateau region. Consequently, more than half grids in the Tibetan Plateau region would have severe risk and more than 75% grids would have moderate risk of ecosystem shifts. In contrast to the high latitudes where soil respiration may increase and soil carbon stocks decrease, the soil carbon stocks in Tibetan Plateau would increase (Supplementary Table S2). It suggests that the increased vegetation carbon input to soil in this region is larger than the loss through soil respiration in a warmer environment. The Γ estimates for all variables are above the moderate risk level for more than 75% of the grids in the temperate humid/semi-humid regions (regions I, II, and IV). Relatively large increases in carbon contained in vegetation, fire carbon, and NPP have caused the large Γ estimates in temperate humid/sub-humid region (Supplementary Table S2). The Γ estimates for carbon stocks are greater than 0.1 for all the grids and Γ estimates for carbon fluxes are greater than 0.1 for about or over half girds in the above three regions. It confirms that with the three major factors considered, change in carbon is the main contributing factor to the high risk of ecosystem shifts.

### Model spread and uncertainty

Figure 7 shows the standard deviation of the Γ (for all variables) caused by all the available GCM-GGVM pairs, GCMs and GGVMs at the end of the 21^st^ century under four RCPs. The model spread is less than 0.1 across the RCPs in the northwest arid region and south part of the tropical and sub-tropical humid region where the risk of ecosystem shifts is generally low. The model spread is generally high in the area where the estimated risk of ecosystem shifts is high. The largest model spread is found in the Tibetan Plateau region. The standard deviation of the Γ estimates is about 0.15 under RCP 2.6, and reaches 0.25 under RCP 8.5. Although most model pairs support a high risk of ecosystem shifts at the Tibetan Plateau region (Supplementary Figure S4), the large standard deviations indicate that there are model pairs supplying a conservative risk assessment.

The uncertainty in Γ estimates arising from the GGVMs is on average approximately twice as large as the uncertainty arising from the GCMs, in line with the finding in Warszawski *et al.*^11^ and Friend *et al.*^30^. The climatic variables from the GCMs were bias-corrected against ground observations in the historical period^18^. The bias-correction may have reduced the spread among the GCMs. The uncertainty arising from the GCMs is largely in the southwest part of the Tibetan Plateau region where ground observations were sparse. The model spread arising from the GCMs is similar across the RCPs, indicating that different emissions and degrees of global temperature increase would have little impact on the uncertainty caused by the GCMs. The uncertainty arising from the GGVMs is largely in the Tibetan Plateau region and temperate regions where the estimated risk of ecosystem shifts is high. Although the pattern of the uncertainty from the GGVMs is similar across the RCPs, the magnitude of the uncertainty is higher under the high emission scenarios than the low emission scenarios. The increased uncertainty along with global temperature and CO_2_ concentration increase is likely due to different model formulations of the fundamental physiological processes in the GGVMs^30^.

## Discussion and Conclusions

**Figure 2:**
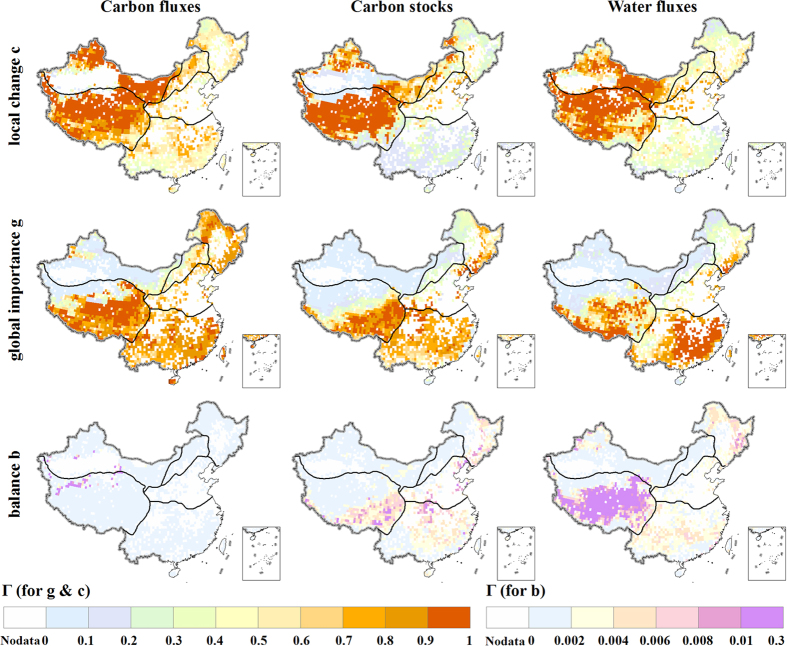
Components (local change, global importance, and balance) of the calculated metric Γ for carbon fluxes (left column), carbon stocks (center column) and water fluxes (right column) at the end of the 21^st^ century under RCP 8.5. Local change (**c**), global importance (**g**), and balance (**b**) are shown at the top, middle and bottom rows, respectively. We generate the maps and integrate them into Fig. 2 using ArcGIS software.

**Figure 3:**
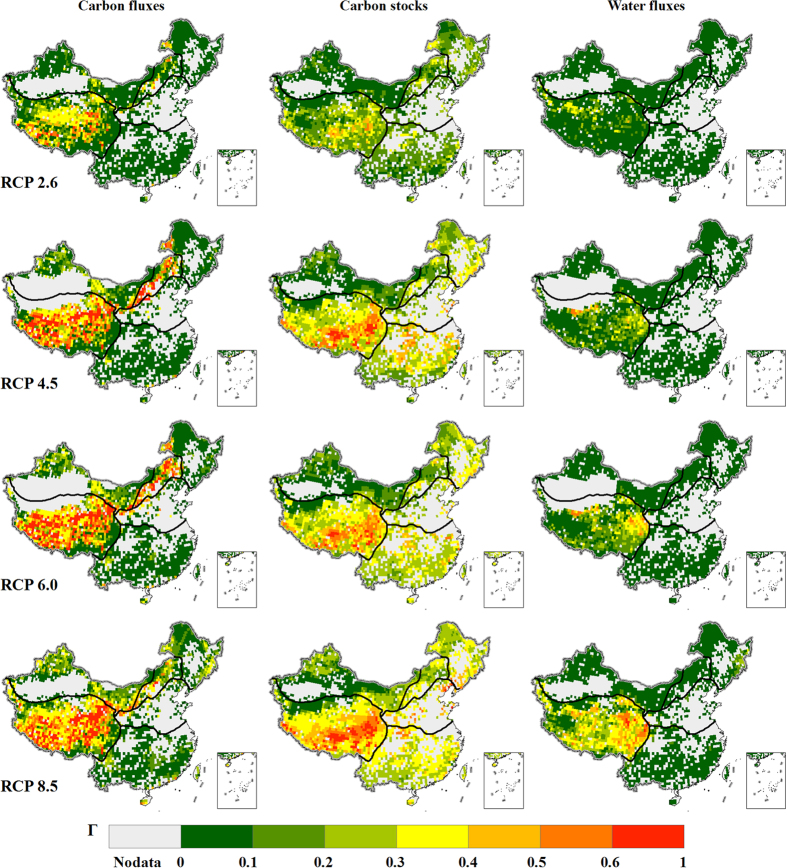
Ecosystem change metric Γ for carbon fluxes (left column), carbon stocks (center column) and water fluxes (right column) at the end of the 21^st^ century under different emission scenarios. The rows from top to bottom show the results under RCP 2.6, RCP 4.5, RCP 6.0, and RCP 8.5, respectively. We generate the maps and integrate them into Fig. 3 using ArcGIS software.

**Figure 4:**
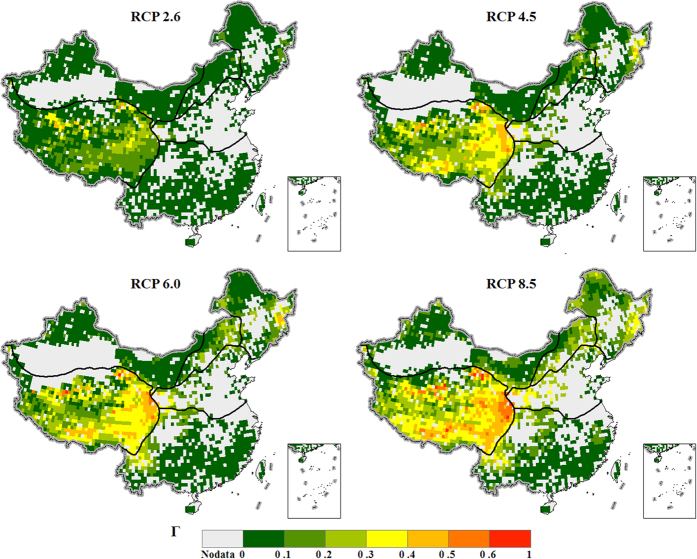
Ecosystem change metric Γ for all variables at the end of the 21^st^ century under four RCPs (RCP 2.6, RCP 4.5, RCP 6.0, and RCP 8.5). We generate the maps and integrate them into Fig. 4 using ArcGIS software.

**Figure 5:**
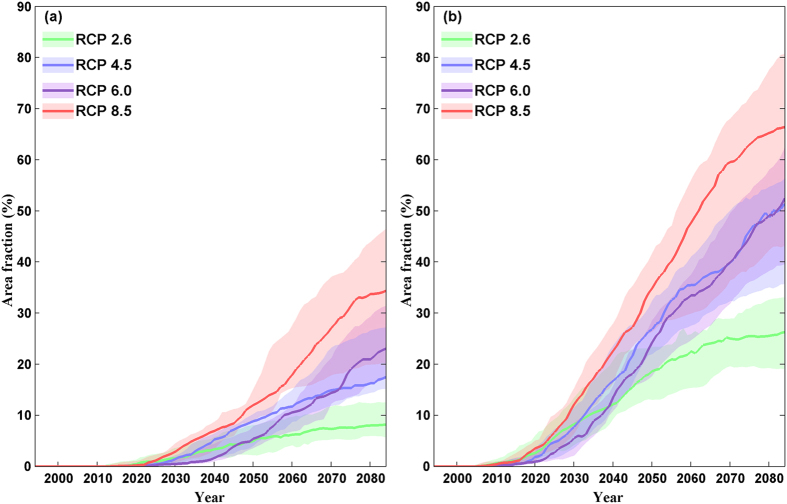
Area fractions of severe risk (**a**) and moderate risk (**b**) of ecosystem shifts from present to the end of the 21^st^ century under four RCPs (RCP 2.6, RCP 4.5, RCP 6.0, and RCP 8.5). The shaded band denotes the inter-quartile range and the solid line shows the median of the GCM-GGVM pairs. We generate the maps and integrate them into Fig. 5 using MATLAB software.

**Figure 6:**
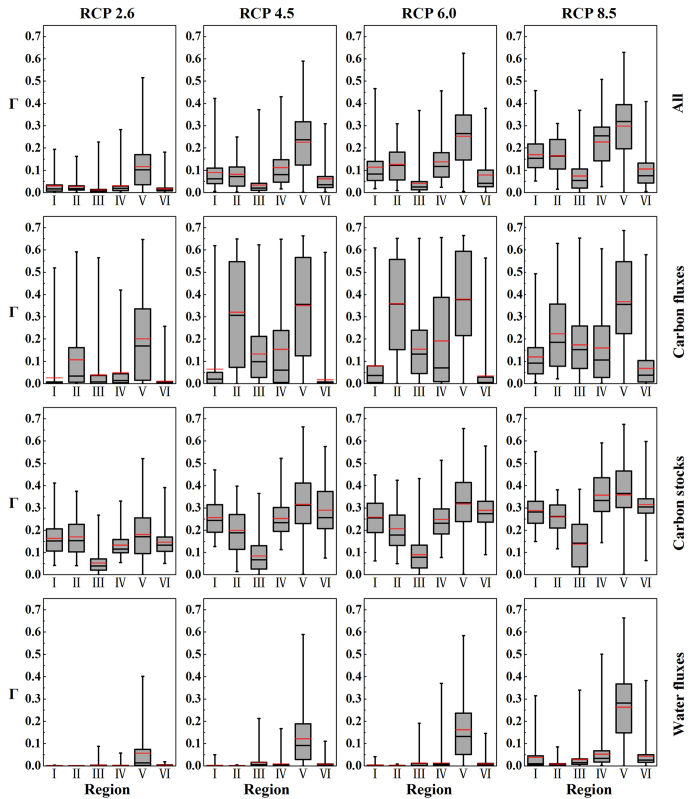
Mean, median and inter-quartile range of the Γ estimates for all variables and variable subsets ‘carbon fluxes’, ‘carbon stocks’, and ‘water fluxes’ in the eco-regions of China at the end of the 21^st^ century under four RCPs (RCP 2.6, RCP 4.5, RCP 6.0, and RCP 8.5). The shaded box shows the inter-quartile range, the vertical line gives the maximum and minimum, the black horizontal line marks the median, and the red horizontal line marks the mean of the Γ estimates in the region. The eco-regions are shown in Fig. 1. We generate the maps and integrate them into Fig. 6 using Origin software.

**Figure 7:**
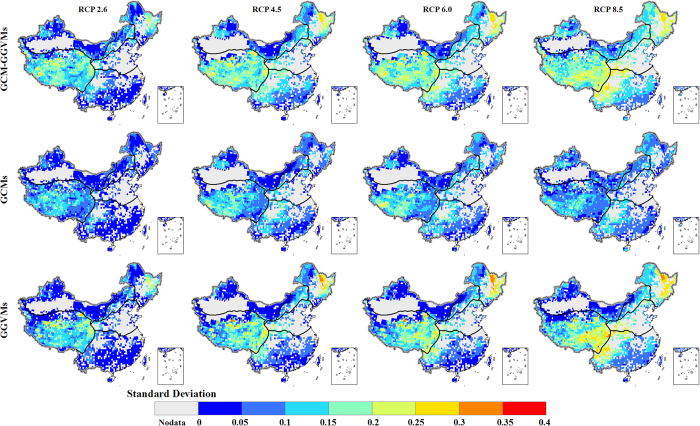
Model spread in the Γ estimates for all variables at the end of 21st century caused by the GCM-GGVM pairs (top row), GCMs (middle row) and GGVMs (bottom row) under four RCPs (RCP 2.6, RCP 4.5, RCP 6.0, and RCP 8.5). We generate the maps and integrate them into Fig. 7 using ArcGIS software.

In this study we assess the risk of ecosystem shifts under climate change in China by calculating an aggregate ecosystem change metric of joint changes in carbon and water fluxes and carbon stocks (Γ). The results show that more than half of the area covered by natural vegetation would face moderate to severe risk of ecosystem shifts at the end of the 21^st^ century under RCP 4.5, RCP 6.0, or RCP 8.5. The Tibetan Plateau is the region with the highest risk of ecosystem shifts in China while the northwest and southern China are the areas with the lowest risk. The high risk in the Tibetan Plateau is likely linked to the fact that carbon stocks and fluxes are most sensitive to temperature changes in the cold area. In the high latitude region, where soil respiration increases and soil carbon stocks decrease in a warmer environment, is more severely affected by global warming^10^. In the Tibetan Plateau region, the soil carbon stocks are not decreasing under a warmer environment because the increased vegetation carbon input to soil caused by warming may compensate the increased loss through soil respiration^31^. Ecosystem risks under climate change have been assessed in China using metrics representing one aspect of ecosystem in the previous studies^6^,^32^. The high-risk regions that this study identified are basically consistent with the previous analyses except for the northwestern China where this study suggested low risk while the previous study^32^ suggested high risk. The previous study^32^ focused on NPP only. However, this study used an aggregated metric which considered multi-dimensions of the ecosystem states. Although the local relative changes in carbon and water are high in the northwestern China (Fig. 2), the overall estimated risk in this region is relatively low (Fig. 3).

With the three major factors considered, the changes in carbon stocks and fluxes are the main contributing factors to the high risk of ecosystem shifts. The change in carbon stocks is generally large with Γ value over 0.3 at the end of the 21^st^ century in all the eco-regions except for the northwest China. The change in carbon fluxes is generally small under low emission scenario, while the change becomes larger under higher emission scenarios but the degree of changes varies for different regions and under different RCPs. The change in water fluxes is generally small in all the eco-regions except for the Tibetan Plateau region. The change in water fluxes has relatively small impacts on ecosystem shifts in the Tibetan Plateau region under low emission scenarios but would have considerable impacts under high emission scenarios.

Although most model pairs agree on an increasing risk of the natural ecosystem in China under all RCPs and support a high risk of ecosystem shifts at the Tibetan Plateau region, there is large uncertainty in the model estimates because of inherent uncertainty of projections from GCMs^33^,^34^ or terrestrial biosphere models^11^,^35^,^36^. The model spread is especially large in the areas where the estimated risk is high. Compared with the GCMs, the GGVMs are the main source of the uncertainty of risk estimates. The uncertainty arising from the GGVMs is higher under the higher emission scenarios than under the lower emission scenarios, suggesting the inherently different responses to the elevated temperature and CO_2_ concentration in the GGVMs.

Given the complexity of terrestrial ecosystem response to climate change, future research should further improve our understanding of climate change impact on multi-processes of the terrestrial ecosystems. It should be noted this study focuses on climate change impacts on natural vegetation only. Other human-induced major drivers of ecosystem change, such as land use change and forest management practices altering the exchange of carbon and water between the atmosphere and terrestrial ecosystem, are not considered in our analysis. The current GGVMs have some fundamental limitations. For example, the constraint of limited supply of nitrogen and other nutrients is generally not well considered in the GGVMs^37^. Nonetheless, this study provides a state-of-the-art multi-model analysis of ecosystem risk under climate change in China and diagnoses the main contributing factors for the risk in the eco-regions. The high-risk areas and driving factors identified in this study could help develop regional strategies for ecosystem conservation to cope with climate change.

## Methods

The ecosystem change metric Γ was calculated following Heyder *et al.*^10^ The metric calculates the difference between an ecosystem state under climate change and the current state. Ecosystem states are characterized as vectors in a multi-dimensional state space by the variables (Supplementary Table S1) simulated by GGVMs, with each dimension representing a specific fluxes change, stock or process variables. The metric Γ is co-determined by the following five dimensions: change in vegetation structure (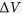)^38^; relative, local change of ecosystem (*c*) (compared to grid cell-specific mean value during the reference period); absolute, global change of ecosystem (*g*) (compared to the global mean value during the reference period); changes in the relative magnitude of key biogeochemical exchange fluxes (*b*) (computed as the angle between certain state vector in future and reference state vector); and change in inter-annual variability (*S*(*x*, *σ*_*x*_)) (a normalized sigmoid function of the ratio to standard deviation in the reference period, and computed for *c*, *g* and *b*). The ecosystem change metric Γ combines these multiple dimensions^10^. The range of value for the above dimensions is 0–1, with 0 being no change and nearly 1 being very strong change (i.e., Γ = 0.98 for a change from mixed forest to desert)^12^. Since the vegetation composition data are unavailable, 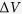 is not included in the calculation. The other three components (c, b and g) were scaled up accordingly in Equation (1) (the factor in the denominator is 3). The exclusion of 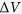
might result in an increased weighting of the other components, but not necessarily reduce or increase the estimated risk.


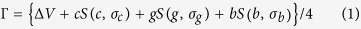


The variables used to compute the metric Γ (Supplementary Table S1) were taken from the simulations of 4 GGVMs – JeDi^39^, JULES^40^,^41^, LPJmL^42^,^43^, VISIT^44^ in ISI-MIP (Supplementary Table S3). These models contain different sub-model types and different parameterizations of carbon and water processes. The dissimilarities of the models and the consequent cautions needed in interpreting the model results are discussed in Friend *et al.*^45^. In order to assess the performance of the GGVMs in China, we compared the model simulated NPP with the remotely sensed NPP estimates in China^46^,^47^. The simulated NPP agrees well with the remotely sensed estimates (Supplementary Figure S5), suggesting the GGVM products may be used in the regional study. The 5 selected GCMs span the core space of relative changes in annual precipitation and changes in annual temperature in China between end of the century and present-day^19^,^48^, indicating that the selected GCMs can appropriately represent the range of possible futures and provide plausible results for future climate in China (Supplementary Figure S6). Table S4 specifies the variables reported by each GGVM, which were included in the calculation. The simulation outputs of the GGVMs were provided from 1971 to 2099. Three GGVMs (JeDi, JULES, and LPJmL) had simulation results for four RCPs (a set of four pathways were produced that lead to radiative forcing levels of 8.5, 6, 4.5 and 2.6 W/m^2^ by the end of the century)^49^ of five GCMs from the Fifth Coupled Model Inter-comparison Project (CMIP5)^50^ but VISIT model had only simulation results for RCP 2.6 and RCP 8.5 of three GCMs (Supplementary Table S5). The model outputs of LPJmL and VISIT were provided on a 0.5° × 0.5° grid, and the model outputs of JeDi and JULES were provided on a 1.25° × 1.85° grid. We assigned the value of a 1.25° × 1.85° grid, to which the central point of a 0.5 degrees grid belong, to the 0.5 degrees grid^51^.

All the GGVMs took into account the CO_2_ fertilization effects. As the land surface was assumed to be covered by natural vegetation only during these model simulations^12^, the ecosystem shifts implied by the GGVMs were only driven by climate change rather than land-use change. Therefore, we focused on the risk of only natural vegetation in this analysis (excluding sandy desert, swamp, and cultivated area) (Fig. 1).

The period of 1981–2010 (denoted as 1995) was used as the reference period to estimate the current state of ecosystem. The reference period was also used in calculation of *c*, *g*, and 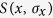. The *g* was calculated based on the data in China, rather than the data over global land surface in the global study. The ecosystem change metric Γ was computed between a future period and the reference period. To ensure that year-to-year variability dose not dominate the signal, a future period was a 30-year period centered on a year from 1995 to 2084. The 30-year moving average was used to denote as the central year of the period. For example, the average of the future period of 2070–2099 was denoted as 2084.

The metric Γ was firstly calculated for each of the variable subset ‘carbon fluxes’, ‘carbon stocks’, or ‘water fluxes’ individually and then was calculated for the variable subset ‘all’ (Supplementary Table S1). If the metric for one variable subset is high, it suggests that the variable subset is a contributing factor to the overall ecosystem shifts. Otherwise, the variable subset has little contribution to the ecosystem shifts. The metric Γ was computed for each GCM-GGVM pair under different RCPs. There were five GCMs and four GGVMs, making a maximum number of model pairs of 20 under each RCP. However, this study used 18 model pairs for RCP 2.6 and RCP 8.5 and 15 model pairs for RCP 4.5 and RCP 6.0 because some model simulations were not available in ISI-MIP (Supplementary Table S5). The multi-model-ensemble median (MMEM) of Γ from the ensemble of all the available GCM-GGVM pairs was calculated.

We considered Γ = 0.3 and Γ = 0.1 as two thresholds for severe and moderate risk, respectively^10^. If the MMEM of the ecosystem change metric Γ for all variable subsets was greater than 0.3 in a future period, the grid was considered to have severe risk of ecosystem shifts. The area fraction of severe or moderate risk was calculated as the area with Γ value greater than 0.3 or greater than 0.1 divided by the total area of natural vegetation in China. In order to illustrate the risk difference within each region, the mean, median and inter-quartile range (range between the 25^th^ and 75^th^ percentiles) of the Γ of the grids were calculated for 6 different eco-regions in China (Fig. 1 and Supplementary Table S6), the boundary of which was adopted from Zheng^30^.

The standard deviation of the Γ estimates from all the available GCM-GGVM pairs was used to quantify the model agreement in assessing the ecosystem shift risk. The model spreads caused by GGVMs and GCMs were evaluated separately. The standard deviation of the Γ estimates from the four GGVMs was firstly calculated for each GCM. The averaged GGVM standard deviations of the five GCMs was then used to assess the model spread caused by GGVMs. Using the same calculation procedure, the averaged GCM standard deviations of the four GGVMs was used to assess the model spread caused by GCMs.

## Additional Information

**How to cite this article**: Yin, Y. *et al.* Risk and contributing factors of ecosystem shifts over naturally vegetated land under climate change in China. *Sci. Rep.* **6**, 20905; doi: 10.1038/srep20905 (2016).

## Supplementary Material

Supplementary Information
