# Supplementary material for: Risk and contributing factors of ecosystem shifts over naturally vegetated land under climate change in China
Source: Sci Rep. 2016 Feb 12;6:20905. doi: 10.1038/srep20905 (PMC4751438; doi:10.1038/srep20905)
Supplement: Supplementary Information [file srep20905-s1.pdf]

Risk and contributing factors of ecosystem shifts over naturally vegetated land under climate change in China

Yuanyuan Yin[1] Qiuhong Tang[1*] Lixin Wang[2] Xingcai Liu[1]

1 Key Laboratory of Water Cycle and Related Land Surface Processes, Institute of Geographic Sciences and Natural Resources Research, Chinese Academy of Sciences, Beijing 100101, China

2 Department of Earth Sciences, Indiana University-Purdue University Indianapolis (IUPUI), Indianapolis IN 46202, USA

*Corresponding author email: tangqh@igsnrr.ac.cn

Contents of this file

Figures S1 to S6
Tables S1 to S6
References

Introduction

This file contains the Supplemental Figure Captions and Table Captions. The references used in those captions have been listed in Reference.

Supplemental Figures

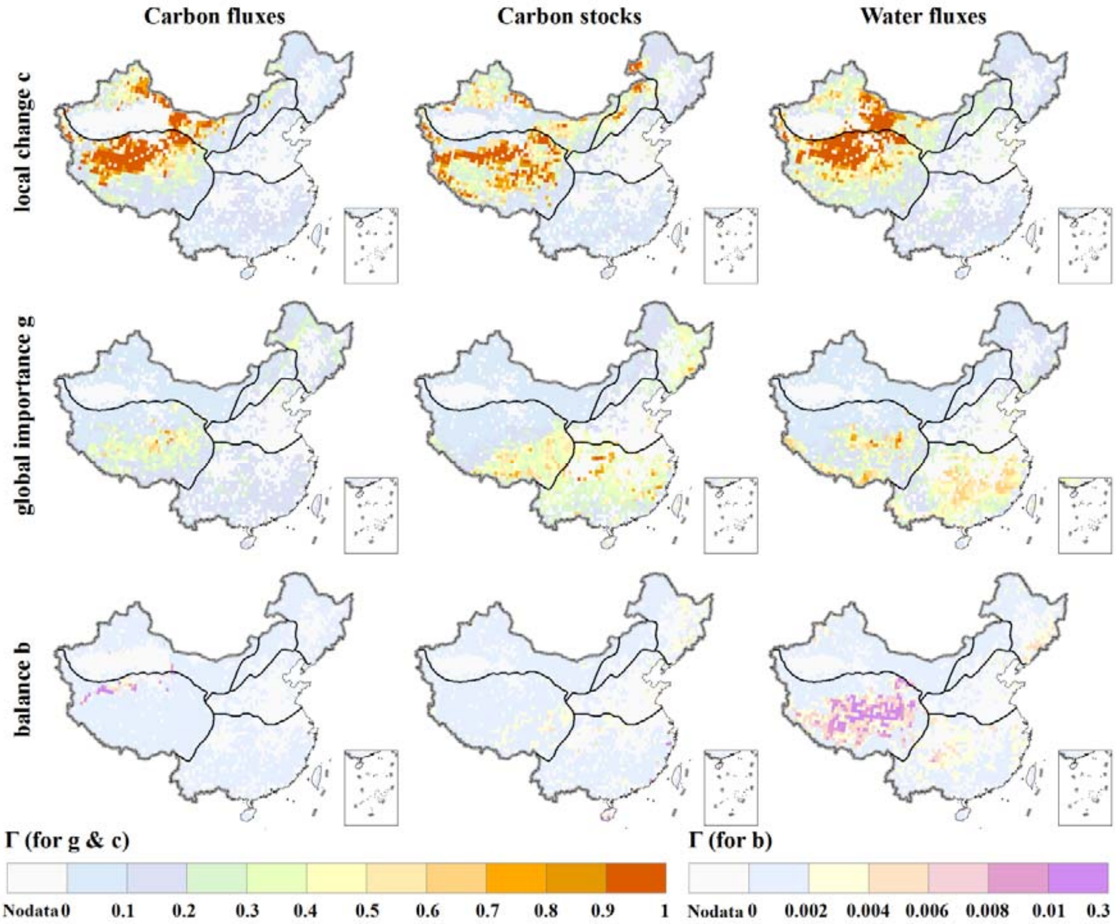

Figure S1 Components (local change, global importance, and balance) of the calculated metric Γ for carbon fluxes (left column), carbon stocks (center column) and water fluxes (right column) at the end of the 21st century under RCP 2.6. Local change (c), global importance (g), and balance (b) are shown at the top, middle and bottom rows, respectively. We generate the maps and integrate them into Figure S1 using ArcGIS software.

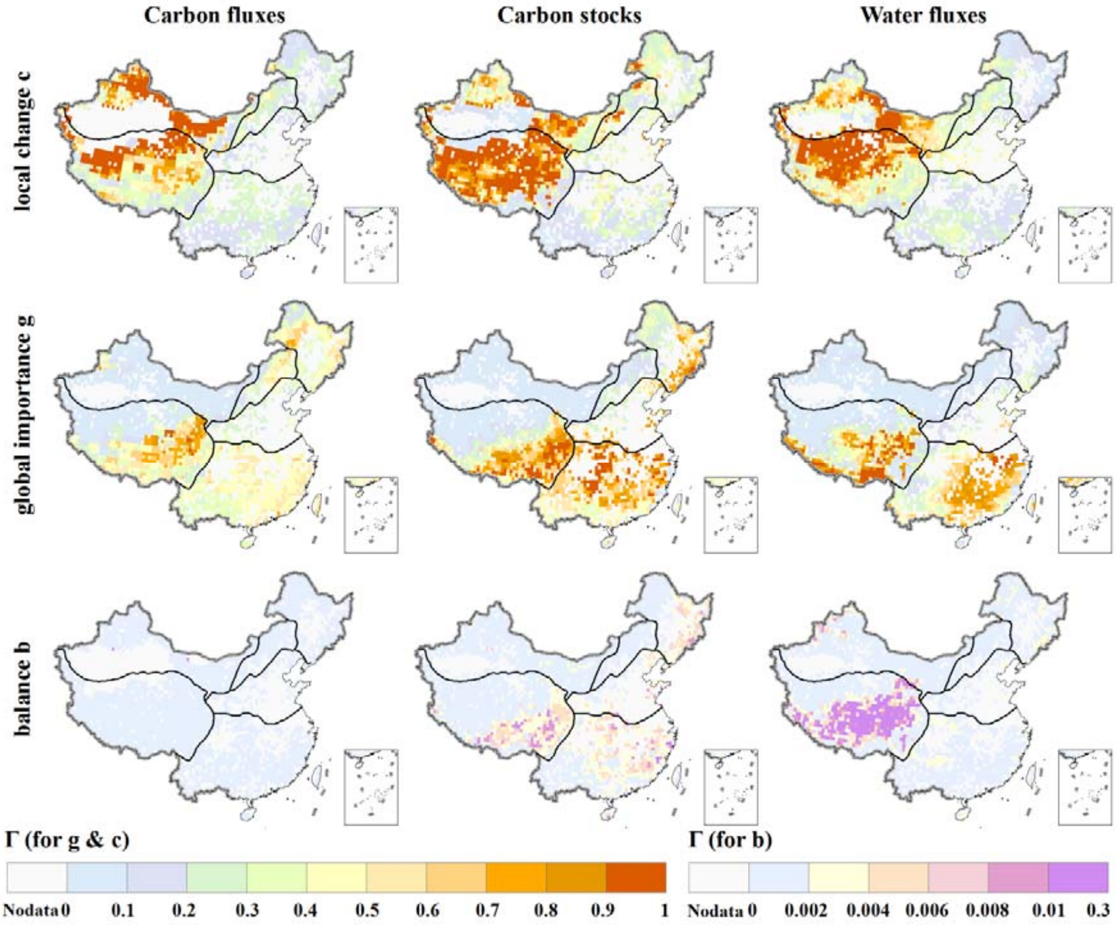

Figure S2 Components (local change, global importance, and balance) of the calculated metric Γ for carbon fluxes (left column), carbon stocks (center column) and water fluxes (right column) at the end of the 21st century under RCP 4.5. Local change (c), global importance (g), and balance (b) are shown at the top, middle and bottom rows, respectively. We generate the maps and integrate them into Figure S2 using ArcGIS software.

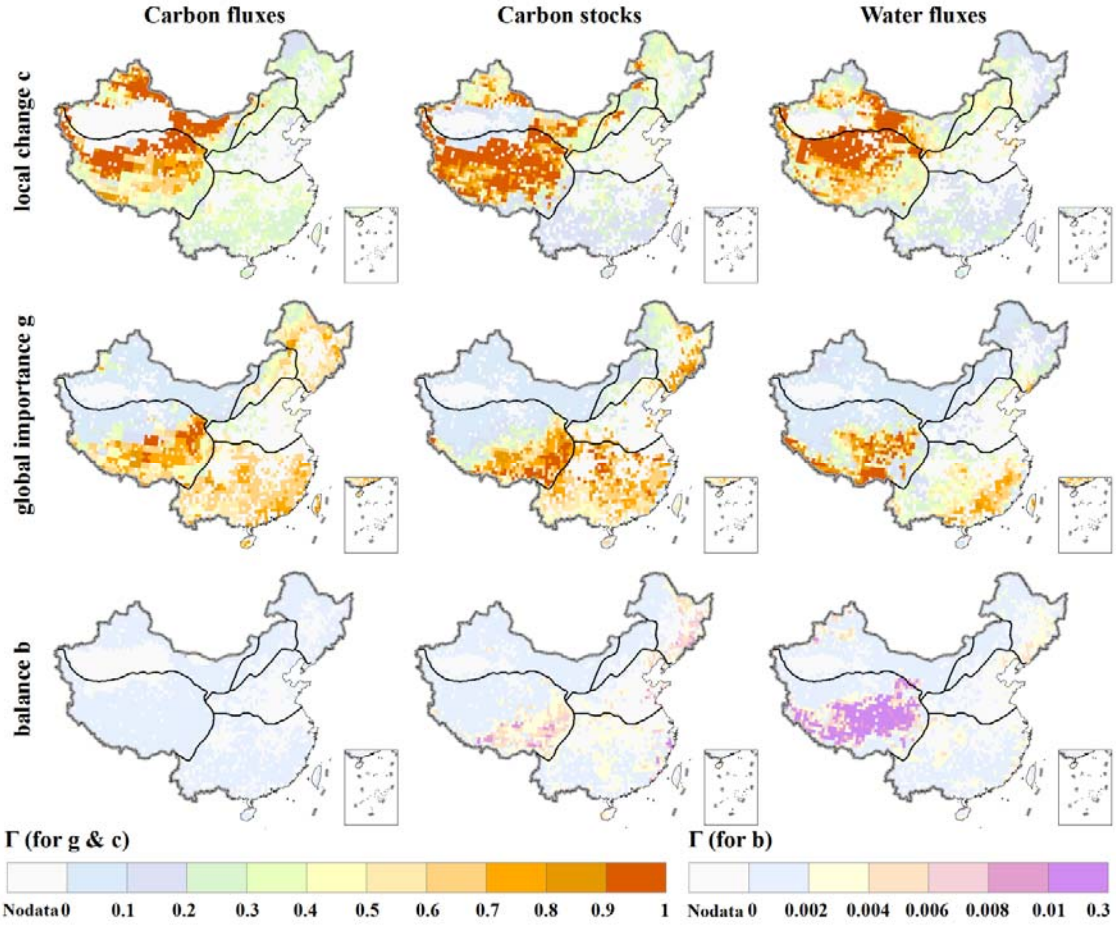

Figure S3 Components (local change, global importance, and balance) of the calculated metric Γ for carbon fluxes (left column), carbon stocks (center column) and water fluxes (right column) at the end of the 21st century under RCP 6.0. Local change (c), global importance (g), and balance (b) are shown at the top, middle and bottom rows, respectively. We generate the maps and integrate them into Figure S3 using ArcGIS software.

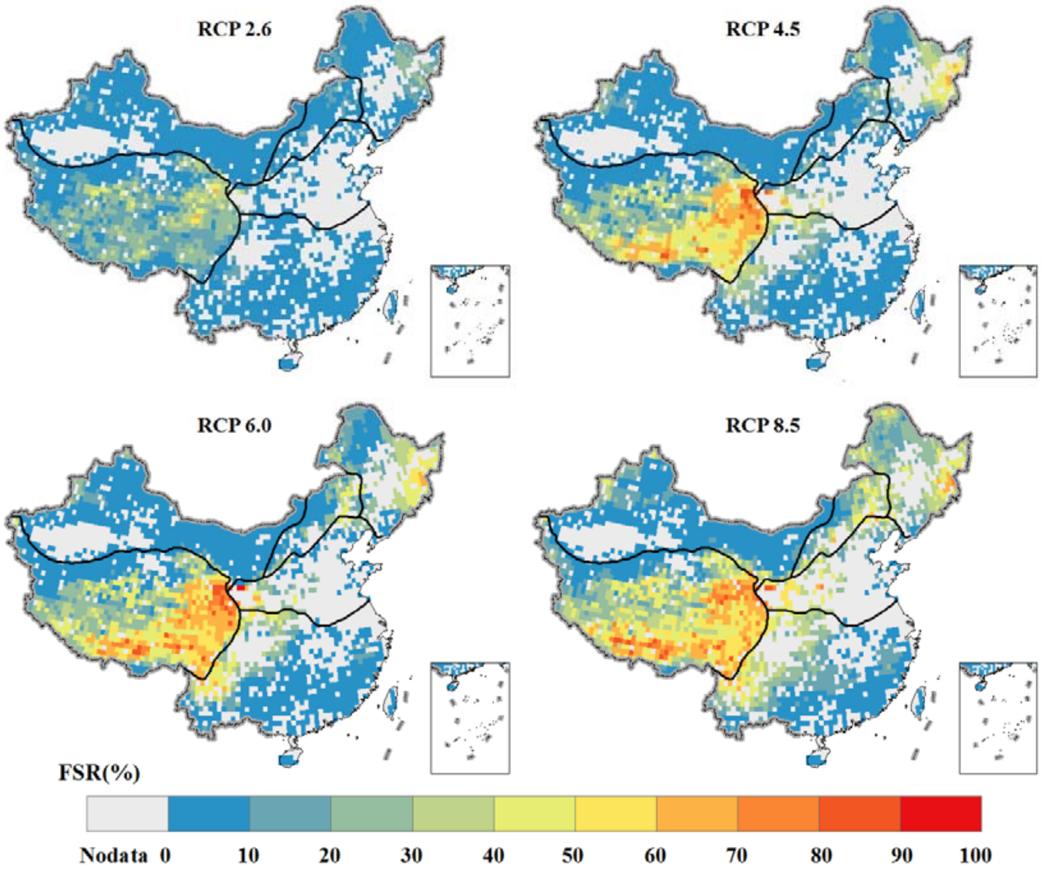

Figure S4 Fraction of severe risk estimates (FSR) at the end of the 21st century under four RCPs. The FSR was calculated as the number of the GCM-GGVM pairs showing severe risk ($\Gamma \geq 0.3$) divided by the total number of the GCM-GGVM pairs to show the model consistency in supporting a severe risk. We generate the maps and integrate them into Figure S4 using ArcGIS software.

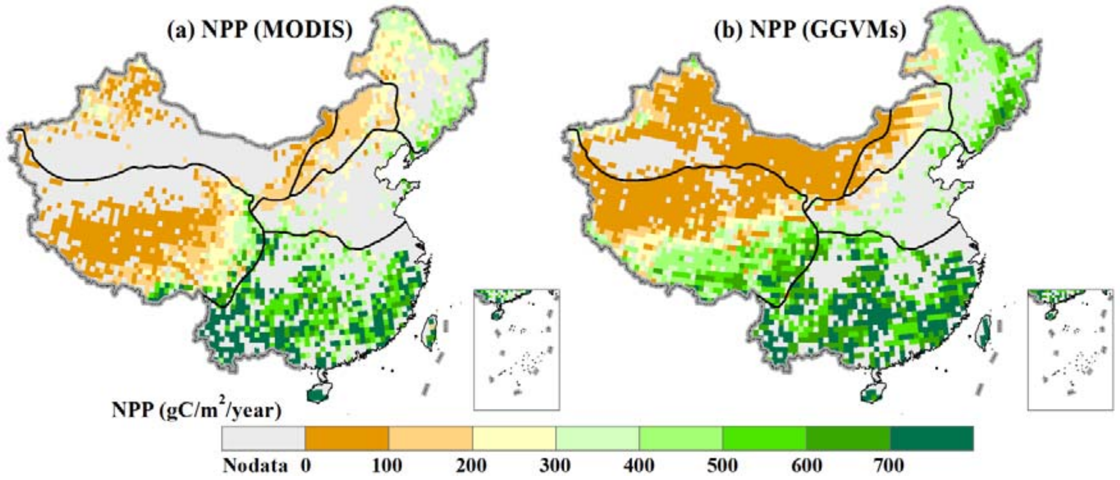

Figure S5 Average annual NPP derived from MODIS (a) and the median of the average annual NPP of GCM-GGVMs (b) during 2000-2005. We generate the maps and integrate them into Figure S5 using ArcGIS software.

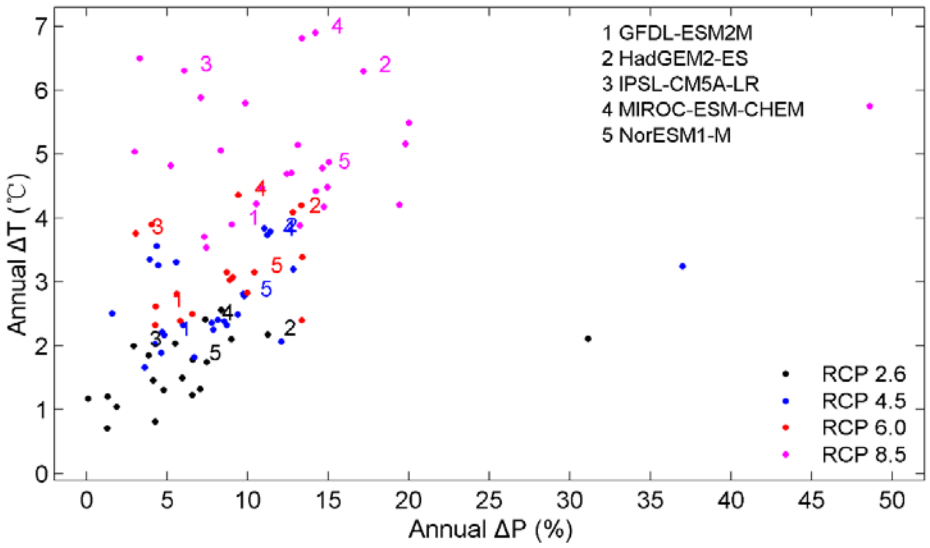

Figure S6 The positions of the five selected GCMs in the space of relative changes in annual precipitation (ΔP) and changes in annual temperature (ΔT) in China between the end of the century (2071-2100) and present-day (1971-2000) from 30 GCMs. The 30 GCMs are bcc-csm1-1, bcc-csm1-1-m, CanESM2, CCSM4, CESM1-BGC, CESM1-CAM5, CMCC-CESM, CMCC-CM, CMCC-CMS, CNRM-CM5, CSIRO-Mk3-6-0, GFDL-ESM2G, GFDL-ESM2M, GISS-E2-H, GISS-E2-H-CC, GISS-E2-R, GISS-E2-R-CC, inmcm4, IPSL-CM5A-LR, IPSL-CM5A-MR, IPSL-CM5B-LR, MIROC5, MIROC-ESM, MIROC-ESM-CHEM, MPI-ESM-LR, MPI-ESM-MR, MRI-CGCM3, NorESM1-M, NorESM1-ME, and HadGEM2-ES. We generate the map using MATLAB software.

Supplemental Tables

Table S1 Variable subsets used for analysis

**Subset**	**Variables**
Carbon fluxes	Net primary production (NPP); fire carbon
Carbon stocks	Carbon contained in vegetation and soil
Water fluxes	Transpiration; evaporation; runoff
All	Carbon fluxes; carbon stocks; water fluxes; soil water content (SWC)

Table S2 Relative change of variables in different eco-regions at the end of the 21st century (2071-2099) compared with the historical period (1981-2010) under RCP 8.5 (%)

**Zone**	**Carbon stocks**		**Carbon fluxes**		**Water fluxes**			**SWC**
	**Carbon_soil**	**Carbon_veg**	**Fire carbon**	**NPP**	**Runoff**	**Evaporation**	**Transpiration**	
Zone Ⅰ	28.08	11.6	10.74	42.74	3.24	-21.47	18.35	-8.89
Zone Ⅱ	15.88	76.53	76.26	43.28	33.29	-18.26	15.08	-8.98
ZoneⅢ	17.49	42.73	123.88	67.43	15.58	-6.37	31.54	-0.88
ZoneⅣ	24.29	56.04	6.09	43.29	28.93	-9.41	15.41	-8.83
Zone Ⅴ	37.69	103.2	82.16	97.28	-1.04	-32.14	42.08	-3.71
ZoneⅥ	9.98	57.9	30.37	53.42	-8.44	-33.62	2.85	-3.95

Table S3 Overview of the GCMs and GGVMs

	**Name**	**Institute**	**References**
GCMs	HadGEM2-ES	Met Office Hadley Centre	*Jones et al.*[1]
	IPSL-CM5A-LR	Institute Pierre-Simon Laplace	*Mignot et al.*[2]
	MIROC-ESM-CHEM	Japan Agency for Marine-Earth Science and Technology, Atmosphere and Ocean Research Institute (The University of Tokyo), and National Institute for Environmental Studies	*Watanabe et al.*[3]
	GFDL-ESM2M	Geophysical Fluid Dynamics Laboratory	*Dunne et al.*[4,5]
	NorESM1-M	Norwegian Climate Centre	*Bentsen et al.*[6]; *Iversen et al.*[7]
GGVMs	JeDi	Max-Planck-Institut für Biogeochemie (Germany)	*Pavlick et al.*[8]
	JULES	Centre for Ecology and Hydrology (UK); Met Office Hadley	*Best et al.*[9];

		Centre (UK); University of Exeter (UK)	*Clark et al.*[10]
	LPJmL	PIK (Germany)	*Rost et al.*[11]; *Bondeau et al.*[12]
	VISIT	National Institute for Environmental Studies (Japan)	*Inatomi et al.*[13]

Table S4 Variables reported by each GGVM, which were included in the calculation of Γ

	**NPP**	**Fire Carbon**	**Carbon_veg**	**Carbon_soil**	**Transpiration**	**Evaporation**	**Runoff**	**SWC**
JeDi	√	--	√	√	√	√	√	√
JULES	√	--	√	√	--	--	√	√
LPJmL	√	√	√	√	√	√	√	√
VISIT	√	√	√	√	√	√	√	√

Note: NPP, Carbon_veg, Carbon_soil and SWC are short for Net Primary Production, Carbon contained in vegetation, Carbon contained in soil and soil water content respectively.

Table S5 Combinations of GCMs, GGVMs and RCPs used in the full model ensembles

	**gfdl-esm2m**				**hadgem2-es**				**ipsl-cm5a**				**miroc-esm-chem**				**noresm1-m**			
RCP	8.5	6.0	4.5	2.6	8.5	6.0	4.5	2.6	8.5	6.0	4.5	2.6	8.5	6.0	4.5	2.6	8.5	6.0	4.5	2.6
JeDi	√	√	√	√	√	√	√	√	√	√	√	√	√	√	√	√	√	√	√	√
JULES	√	√	√	√	√	√	√	√	√	√	√	√	√	√	√	√	√	√	√	√
LPJmL	√	√	√	√	√	√	√	√	√	√	√	√	√	√	√	√	√	√	√	√
VISIT	√	--	--	√	√	--	--	√	√	--	--	√	--	--	--	--	--	--	--	--

Table S6 Eco-regions and climatic indices[14]

**Serial**	**Eco-regions**	**Typical climatic indices**	
		**Days of accumulated temperature >10 °C**	**Aridity index**
Ⅰ	Cold temperate humid region	<170	<1.5
Ⅱ	Temperate humid/sub-humid region	100-220	1.5-4
Ⅲ	Northwest arid region	100-220	>4
Ⅳ	Warm temperate humid/ sub-humid region	171-220	<1.5
Ⅴ	Tibetan Plateau region	<180	--
Ⅵ	Tropical and sub-tropical humid region	>221	<1

References

[1] Jones, C. D. et al. The HadGEM2-ES implementation of CMIP5 centennial simulations. *Geosci. Model Dev.* **4,** 543-570 (2011).

[2] Mignot, J., & Bony, S. Presentation and analysis of the IPSL and CNRM climate models used in CMIP5. *Clim. Dynam.* **40,** 2089, doi: 10.1007/s00382-013-1720-1 (2013).

[3] Watanabe, S. et al. MIROC-ESM 2010: model description and basic results of CMIP5-20c3m experiments. *Geosci. Model Dev.* **4,** 845-872 (2011).

[4] Dunne, J. P. et al. GFDL's ESM2 global coupled climate–carbon earth system models, part 1: physical formulation and baseline simulation characteristics. *J. Climate.* **25,** 6646-6665 (2012).

[5] Dunne, J. P. et al. GFDL's ESM2 global coupled climate–carbon earth system models, part 2: carbon system formulation and baseline simulation characteristics. *J. Climate.* **26,** 2247-2267 (2013).

[6] Bentsen, M. et al. The Norwegian Earth System Model, NorESM1-M – part 1: description and basic evaluation of the physical climate. *Geosci. Model Dev.* **6,** 687-720 (2013).

[7] Iversen, T. et al. The Norwegian Earth System Model, NorESM1-M – part 2: climate response and scenario projections. *Geosci. Model Dev.* **6,** 389-415 (2013).

[8] Pavlick, R., Drewry, D. T., Bohn, K., Reu, B., & Kleidon, A. The Jena Diversity-Dynamic Global Vegetation Model (JeDi-DGVM): a diverse approach to representing terrestrial biogeography and biogeochemistry based on plant functional trade-offs. *Biogeosciences* **10,** 4137-4177 (2013).

[9] Best, M. J. et al. The Joint UK Land Environment Simulator (JULES), model description – part 1: energy and water fluxes. *Geosci. Model Dev.* **4,** 677-699 (2011).

[10] Clark, D. B. et al. The Joint UK Land Environment Simulator (JULES), model description – part 2: carbon fluxes and vegetation. *Geosci. Model Dev.* **4,** 701-722 (2011).

[11] Rost, S. et al. Agricultural green and blue water consumption and its influence on the global water system. *Water Resour. Res.* **44,** 1-17 (2008).

[12] Bondeau, A. et al. Modelling the role of agriculture for the 20th century global terrestrial carbon balance. *Global Change Biol.* **13,** 679-706 (2007).

[13] Inatomi, M., Ito, A., Ishijima, K., & Murayama, S. Greenhouse gas budget of a cool-temperate deciduous broad-leaved forest in Japan estimated using a process-based model. *Ecosystems* **13,** 472-483 (2010).

[14] Zheng, D. A study on the eco-geographic regional system of China. Food Agricultural Organization (FAO), FRA 2000: Global ecological zones mapping. Cambridge, United Kingdom (1999).
